# Competing coexisting phases in 2D water

**DOI:** 10.1038/srep25938

**Published:** 2016-05-17

**Authors:** Jean-Marc Zanotti, Patrick Judeinstein, Simona Dalla-Bernardina, Gaëlle Creff, Jean-Blaise Brubach, Pascale Roy, Marco Bonetti, Jacques Ollivier, Dimitrios Sakellariou, Marie-Claire Bellissent-Funel

**Affiliations:** 1 Laboratoire Léon Brillouin, CEA, CNRS, Université Paris-Saclay, CEA Saclay, 91191 Gif-sur-Yvette Cedex, France; 2 ICMMO, (UMR CNRS 8182), Univ. Paris-Sud, Université Paris-Saclay, 91405 Orsay, France; 3 Synchrotron SOLEIL, L’Orme des Merisiers, BP 48, Gif-sur-Yvette Cedex F-91192, France; 4 SPEC, CEA, CNRS, Université Paris-Saclay, CEA Saclay, 91191 Gif-sur-Yvette Cedex, France; 5 Institut Laue Langevin, 38042 Grenoble CEDEX, France; 6 NIMBE, CEA, CNRS, Université Paris-Saclay, CEA Saclay, 91191 Gif-sur-Yvette Cedex, France

## Abstract

The properties of bulk water come from a delicate balance of interactions on length scales encompassing several orders of magnitudes: i) the Hydrogen Bond (HBond) at the molecular scale and ii) the extension of this HBond network up to the macroscopic level. Here, we address the physics of water when the three dimensional extension of the HBond network is frustrated, so that the water molecules are forced to organize in only two dimensions. We account for the large scale fluctuating HBond network by an analytical mean-field percolation model. This approach provides a coherent interpretation of the different events experimentally (calorimetry, neutron, NMR, near and far infra-red spectroscopies) detected in interfacial water at 160, 220 and 250 K. Starting from an amorphous state of water at low temperature, these transitions are respectively interpreted as the onset of creation of transient low density patches of 4-HBonded molecules at 160 K, the percolation of these domains at 220 K and finally the total invasion of the surface by them at 250 K. The source of this surprising behaviour in 2D is the frustration of the natural bulk tetrahedral local geometry and the underlying very significant increase in entropy of the interfacial water molecules.

In bulk water, local energetics and long-range connectivity come along with a very specific local three-dimensional (3D) tetrahedral organization of the hydrogen bond (HBond) network (Fig. 1a). Altogether these properties drive the numerous so-called “water anomalies”^1^,^2^, for example, the apparent power-law divergence of thermodynamical (thermal expansivity, specific heat) and dynamical (self-diffusion coefficient) quantities toward a singular region of the phase diagram at *T*_*s*_ ≈ 228 K and *P*_*s*_ ≈ 100 MPa. If proven true, the existence of a Low Temperature Critical Point of water (LTCP)^1^ that would perfectly explain these divergences would lead to a revolutionary re-interpretation of the physics of water. Nevertheless, as the LTCP temperature lays in-between the homogenous nucleation temperature (235 K) and the crystallization temperature of the glassy forms of water (150 K), the properties of the bulk liquid water in the thermodynamic conditions of the LTCP have been so far unreachable to experiments.

Since it is a notorious way to avoid nucleation, nanometric confinement of water is currently intensively used to probe the liquid phase in this 150 to 235 K “No man’s land”^2^. The idea is to exalt the Gibbs-Thomson effect (i.e. the depression of the melting point, see SI Text 1) by confinement of water in porous structure with extremely narrow pores in the nanometer range. Whatever the nature of the surface of the porous network (polar, hydrophilic or hydrophobic) the restriction of the accessible space induced by confinement has indeed important structural, dynamical and thermodynamical consequences.

Transport of water molecules in Carbon NanoTubes (CNT), can be cited as an emblematic example^3^ of how the frustration of the natural 3D tetrahedral organization of the hydrogen bond network by confinement profoundly modifies the water physical properties: when trapped inside CNT, water is found to flow-up three orders of magnitude faster than predicted by the continuum hydrodynamics picture. This is the consequence of the peculiar organization of the water hydrogen bond network imposed by the restricted radial volume of the tube. In a 1.4 nm diameter CNT, as no interaction with the graphenic CNT internal structure is possible^4^, the water molecules form a 1D central chain surrounded by a corona of other water molecules. The chain-water molecules, experiencing only two HBonds (instead of on average 3 to 4 in bulk), they lay in a very metastable state showing an extremely high mobility: they can adopt a liquid-like behavior at temperatures as low^5^ as 150 K.

As it has been reported even in the case of hydrophilic surface interaction, such a hindrance of ice formation has actually been proposed^6^ as a ubiquitous phenomenon for 1D confinement in the nanometer diameter pore range. Molecular Dynamics (MD) simulations data have shown that, even when the surface locally imposes a crystal orientational order, the formation of unfreezable surface water is controlled by the cylindrical pore curvature that induces spontaneous positional disorder preventing the formation of a long range crystalline HBond network.

Confinement of water within a 1D hydrophilic system can also show low temperature interesting features on a dynamical point of view: the self-diffusion coefficient of water confined in MCM41 (cylindrical pore radius of 1.4 nm) follows Arrhenius dependence at low temperature but at 220 K adopts a Vogel–Fulcher–Tammann (VFT) behavior^7^. Using a terminology reminiscent of the glass physics, one talks of a Fragile to Strong Transition (FST). Such a FST had actually been predicted^8^ as a sudden change of the bulk water structure, to explain an apparent discrepancy: as sensed via thermodynamic methods, bulk water near its melting point is the most *fragile* liquid while it is the *strongest liquid* when probed by kinetics techniques.

Biophysics is another important class of scientific issues where low dimension water is at play. Compared to the perfect 1D situation of pure hydrophobic CNT and pure hydrophilic MCM41, water at the surface of protein is a much more complex situation though, as a protein surface is a complex arrangement of hydrophilic and hydrophobic adjacent regions showing two dimensional (2D) roughness and specific heterogeneous dynamics. It is now recognized that a complex interplay of protein/interfacial water HBond dynamics at the protein surface is responsible for a 220 K dynamical transition^9^ above which the proteins can recover their enzymatic or photosynthetic functions. Unexpected interesting phenomena involving 2D water have also been suggested. For example, MD simulations show that a water monolayer formed onto a surface designed with specific charge quantity and distribution can induce competition between Coulomb interactions and thermal fluctuations to turn the water monolayer into a hydrophobic surface^10^. On a more general and technical point-of-view, the two dimensional water is of a high relevance in all the fields where interfacial water is at play: geology, cement technology^11^ and nuclear waste management^12^. It has been indeed recently proposed^13^ that, the self-diffusion coefficient of water, one of the key components controlling the long range water transport properties a central issue common to all these porous systems, is at first order ruled by the sole quantity of interfacial water.

In this paper, we go deeper in the behavior of the HBond network under reduced dimensionality and focus on the physics of water in two dimensions (2D). This topology can be, for example, obtained by water vapour adsorption as a monolayer onto the surface of Vycor, a hydrophilic porous silica glass (Fig. 1b). As the Vycor surface is uniformly covered with silanol (Si-OH) groups, water molecules interact with the neighbouring water molecules, but also with the surface, only through HBonds. This is indeed a key condition to probe the sole influence of the dimensionality on the HBond energetics and connectivity, and discard any other specific interaction with the surface. We indeed consider that the HBond engaged with a silanol OH is similar to the one with another water molecule.

The dynamics of interfacial water at the surface of Vycor has already been analysed in details by neutron diffraction and quasi-elastic neutron scattering. Here, we combine specific heat measurements, Far and Mid Infra-Red (FIR & MIR) spectroscopy, Magic-Angle Spinning Solid-State Quadrupolar NMR (SS-NMR) and Quasi-Elastic Neutron Scattering (QENS) to experimentally investigate the physical behaviour of this Interfacial Water (IW) over a broad temperature range (100–300 K).

Instead of addressing an *average* water dynamics, we gain considerable physical insight by considering separately the rotational and translational modes^6^ to the water molecules overall dynamics. While QENS probes both the rotational and the translational contributions, SS-NMR focuses on the sole rotational contribution and also allows identifying strong dynamical heterogeneities.

## Results

### Compared to ice, interfacial water shows a large specific heat suggesting specific dynamical modes at low temperature

Nanometric confinement of molecular fluids is a classical route to stabilize metastable states by achieving the frustration of the bulk natural fluctuations and/or phase transitions. Figure 2 illustrates how confinement can lead to a dramatic downshift of the water phase diagram: the clear endothermic signal with onset at 250 K in fully hydrated Vycor (25 wt% i.e. the 50 Å characteristic size of Vycor pores are filled with water) is the signature of ice melting. Next to this 23 K depression of the melting point compared to the bulk situation (the so called Gibbs-Thomson effect^14^ and see SI text 1), it is noteworthy how larger is the specific heat (*C*_*P*_) of water as a monolayer in Vycor compared to bulk ice or fully hydrated Vycor. This heat capacity excess is a strong evidence of the large mobility of interfacial water compared to that of ice (in bulk or confined within Vycor). Neutron scattering experiments have indeed already revealed^15^ the underlying dynamical modes at this origin of this mobility (see SI text 2). Also, according to the statistical physics approach, this large *C*_*P*_ values suggest large entropy fluctuations (*C*_*P*_ ~ <*δS*^*2*^>).

### Infra-Red spectra evidence phases and/or dynamical transitions in the 160, 220 and 250 K regions

The temperature dependence of MIR and FIR absorbance spectra of interfacial water from 120 to 300 K are shown in Fig. 3.

In each case, the reference used for calculating the absorbance is the dry sample before hydration and at the same temperature. The MIR and FIR data show a common trend: depending on the temperature, the spectra of 2D-water resemble the one of the LDA amorphous ice or liquid water but are very different from the one of hexagonal ice. While indirect, this is nevertheless evidence that the structure of interfacial water is not crystalline.

The intermolecular OH stretching band (also referred as the connectivity band^17^) in the 100–350 cm^−1^ region is shown in Fig. 3a. This mode is a combination of intermolecular hydrogen bond O-H····O stretching and bending related to clusters of water molecules. Since this mode only involves small protons displacements, this excitation is poorly detected in inelastic incoherent neutron scattering so that this FIR derived information is a real asset in the scope of the present paper.

In order to follow the changes of intensity of the connectivity band, the region between 106 cm^−1^ and 236 cm^−1^ was integrated and a background corresponding to the contribution of the libration band, starting at 250 cm^−1^, was subtracted. The result of this subtraction and the related difference-band integration are presented in Fig. 3c. It shows that the connectivity intensity experiences only few changes between 50 K and the 150 K region, but that it strongly decreases above 150–160 K. This indicates a modification of the connectivity of the HBond network around 160 K. This mode is not detected anymore above 175 K.

MIR spectra of 2D water (Fig. 3b) can be decomposed in three distinct intramolecular bands: 1910–2510 cm^−1^ related to a mixing of bending and libration modes, and 2912–3149 cm^−1^ and 3183–3356 cm^−1^ two bands related to the OH stretching mode. A monolayer of water molecules on Vycor produces an OH stretching mode at 3230 cm^−1^. The position of this peak reveals that the HBonds at play in the system are significantly stronger than in bulk liquid water. A numerical estimate of the enthalpy of these HBonds will be given below.

Measurements as a function of temperature between 75 and 300 K show that the frequency of the stretching band maximum shifts from lower to higher frequencies as the temperature increases. This is perfectly in line with previous deep inelastic neutron scattering measurements^18^. As shown by IR study of ice VII under pressure^19^, a shift of the intra-molecular stretching OH towards higher energy is correlated with a decrease of the H-Bond strength. Two changes of slope at 160 K and 250 K suggest that there are two dynamical transitions in the H-bond strength.

The MIR spectra also carry critical information about the sample characterization and in particular its temperature stability: the silanol groups (Si-O-H) of the Vycor matrix appear at 3700 cm^−1^ as a negative band. This shows that the number of free silanol groups of the dry Vycor significantly decrease upon water adsorption. The fact that the band remains stable shows that for all temperature, the water molecules remain bonded to the surface and no sublimation or lyophilisation occurs at any temperature.

### Solid State Quadrupolar NMR reveals a strong dynamical heterogeneity and the temperature dependence of a liquid-like and a solid-like phase

Since both neutron scattering and IR spectroscopy probe the ensemble average of the water molecules dynamics, no information is available about any possible dynamical heterogeneity. ^2^H SS-NMR spectra of water as a monolayer on Vycor have been recorded over a large temperature range (Fig. 4). At the lowest temperatures, all these NMR spectra show two very distinct contributions: a broad patterned doublet (*C*_*QQ*_ ≈ 220 ± 10 kHz for the temperature range 100 K–220 K, *C*_*QQ*_ ≈ 190 ± 10 kHz above 220 K) and a narrower signal (≈20 kHz FWHM). The first signal demonstrates the presence of immobilized water molecules (*solid-like*), while the second one, assigned to water molecules experiencing a significant degree of reorientational mobility, evidences a *liquid-like* water fraction, *f*, within the sample. In addition, as it is sensitive only to the reorientational dynamics, quadrupolar NMR can be used to specifically probe the water molecule rotational contribution to the water dynamics.

At all the investigated temperatures, from 100 to 270 K, the central line is well accounted for by a Lorentzian line and *f* can be simply evaluated by considering the relative integrated intensity of this Lorentzian (Fig. 4b). The onset of the rotational motion is clearly detected around 160 K and is followed by a sigmoidal increase with a characteristic temperature around 220 K. These two temperatures correspond to the onset of dynamical rotational events detected by neutron scattering^15^. Nevertheless, the NMR results bring into play important new information: the fraction of population experiencing the rotational dynamics is strongly temperature dependent *i.e.* the dynamics is highly heterogeneous.

### A mean-field percolation model to account for the connectivity of the hydrogen bond network

Hydrogen bond rules the properties of water on two very different length scales: at the local scale, a pure energetic term defines if the bond is formed or not. In bulk, a water molecule can be engaged in 0, 1, 2, 3 or 4 HBonds with its neighboring molecules. The directionality of the O-H---O vector (see SI text reference/note 1) and the O O distance are there the key parameters. At a significantly larger scale, encompassing tens of water molecules, the relevant parameter is not energetics but the connectivity of the hydrogen bond network. Stanley and Teixeira^20^ have derived a random-bond percolation model to interpret the unusual properties of bulk water.

Let’s consider *p* as the probability, to form a hydrogen bond. Symmetrically, *(1–p)* is the probability that a possible HBond, chosen at random, is broken. In bulk, the water molecules can then be classified in 5 groups *f*_*i*_ (*i* = 0, 1…4), where *i* defines the number of intact HBonds engaged by a given molecule. This statistics is strongly affected by the temperature (below 273 K, the probability to have 4 stable HBonds is close to 1).

In this paper, water molecules have a strong interaction with the Vycor surface. HBonds between the interfacial water molecules and the numerous silanol groups at the surface of Vycor can therefore be considered as rather stable. Then by respect to the bulk situation, in the interfacial situation, the statistics is modified^21^: *f*_*0*_ = 0 (a molecule has always a HBond engaged with a Vycor surface silanol group). The statistics of the *f*_*i*_ classes simply follows the binomial distribution of the remaining three possible HBonds:


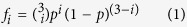


**Figure 1:**
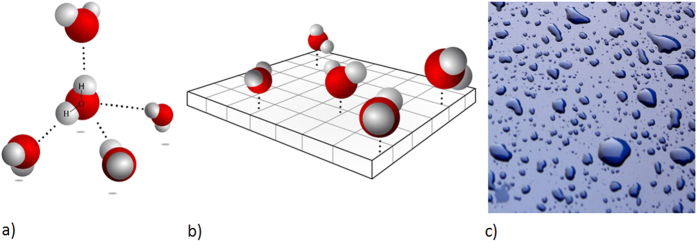
(**a**) In bulk liquid water, a molecule is connected by HBonds (dotted lines) to an average of slightly less than four molecules. We represent here the perfect tetrahedral geometry found in hexagonal ice (after Wikipedia commons). (**b**) In this paper, water is adsorbed as a monolayer (6 wt%) onto the surface of Vycor, a porous hydrophilic silica glass whose surface is uniformly covered with silanol (Si-OH) groups. Vycor surface presents 18 silanol groups by square nanometer. This sketch is a realistic graphical representation of the microscopic structure of the 2D-water we consider: the water molecules form a single monolayer. In this topology, the ideal tetrahedral arrangement shown in (**a**) is lost and each water molecule interacts with the neighboring molecules with distorted HBonds (not shown). All the water molecules are nevertheless always HBonded (dotted lines) to a surface silanol. For clarity, these groups are not explicitly represented, but each basal square on the sketch stands for the specific surface of a single silanol. (**c**) Artist view (image courtesy of Robby_m, rgbstock.com) of the structure of interfacial water at the mesoscopic scale this paper concludes to: interfacial water is a heterogeneous structure where high density monolayer patches (dark blue) coexist with low density ones (light blues). The temperature dependence of this structure on the whole investigated temperature range is shown in Fig. 6.

The molecules belonging to the *f*_*4*_class are not randomly distributed (see SI text 3) but instead form “patches” (represented as dark blue zones in Fig. 1c), where all the water molecules form 4 hydrogen bonds. In bulk, Molecular Dynamics Simulations have shown that the sharp increase in the fraction of four-coordinated molecules in supercooled liquid water explains its anomalous thermodynamics and also controls the rate and mechanisms of ice formation^22^.

The statistical approach can be developed further to evaluate G*(p), the total number, and S*(p), the average size of the patches of four HBonded molecules (see SI text 4):


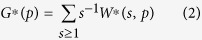



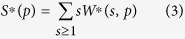



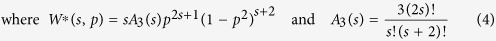


W*(s, p) is the weight fraction of molecules part of an *s-*site network.

Compared to the overall HBond global network, the high connectivity within these domains induces a local lower density (ice floats on liquid water!) and entropy. As the temperature is lowered, the probability of forming Hbonds increases. The size and the number of the *f*_*4*_ patches increase accordingly and hence the entropy fluctuations.

### The self-diffusion coefficient as measured by QENS can be used to tune the temperature dependence of *p*, the probability to form a hydrogen bond

To tune the temperature dependence of *p*, we use the temperature dependence of *D*_*s*_, the self-diffusion coefficient (Fig. 5a) of IW as measured at the local scale by incoherent QENS.

The self-diffusion coefficient is directly controlled by the fraction of immobile water molecules (see SE1 in SI text 5):


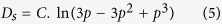


where C is a constant. The *D*_*s*_ data Fig. 5a are fairly well described (Fig. 5b) with a simple temperature dependence *p(T)* *=* α − β*T with *α* *=* 1.51 and*β* *=* 3.65 10^−3^ K^−1^. Using *p(T)* as an input the temperature dependence of S* and G* can be determined: S*, the size of the patches of 4 HBonded molecules reaches a maximum at 220 K, while G* the number of these high density patches is maximum at 240–250 K (Fig. 5). These two predicted temperature crossovers are perfectly in tune with the transitions experimentally detected in NMR and QENS.

### Estimate of thermodynamical quantities: HBond enthalpy and entropy of 2D water

While this simple analytical approach is able to reproduce, the temperatures of dynamical changes experimentally observed, at this stage, key information when it comes to phase transitions are still missing, in particular thermodynamics quantities like entropy and enthalpy.

These quantities can be introduced if one considers the water connectivity as the result of the dynamical equilibrium between broken and intact Hbonds^23^:


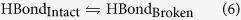


The equilibrium constant writes:


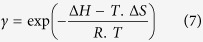


where Δ*H* and Δ*S* are the enthalpy and entropy of an HBond and *R* = 8.31 J/K/mol.

The fraction of broken bond is:


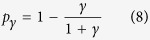


**Figure 2:**
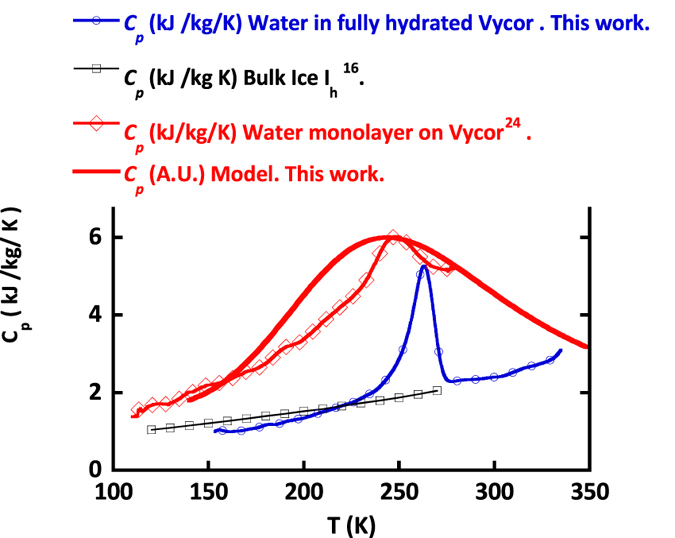
Specific heat (*C*_*p*_) measurement of water confined in fully hydrated (25 wt%) and monolayer hydrated (6 wt%) Vycor (the dry Vycor contribution has been subtracted). Specific heat of bulk hexagonal ice^16^ is given for reference. The very significant specific heat excess of 2D water compared to bulk ice of Vycor confined ice indicates a large mobility of interfacial water compared to ice (bulk or confined within Vycor). The *C*_*p*_ derived from the percolation model presented here is shown as a full thick red line.

Values Δ*H* = 13 kJ/mol and Δ*S* = 48 J/mol/K (Fig. 5b) account for the linear temperature dependence of the fraction of intact interfacial HBonds estimated from the self-diffusion coefficient. Compared to bulk water (Δ*H*_*Bulk*_ = 9.80 kJ/mol and Δ*S*_*Bulk*_ = 21.6 J/mol/K), this clearly shows the excess of entropy, and hence the increased disorder induced by the low dimension of the 2D topology. This large entropy value comes along with large entropy fluctuations as suggested by the large *Cp* value (*C*_*P*_ *~* *<δS*^*2*^*>)* shown on Fig. 2.

**Figure 3:**
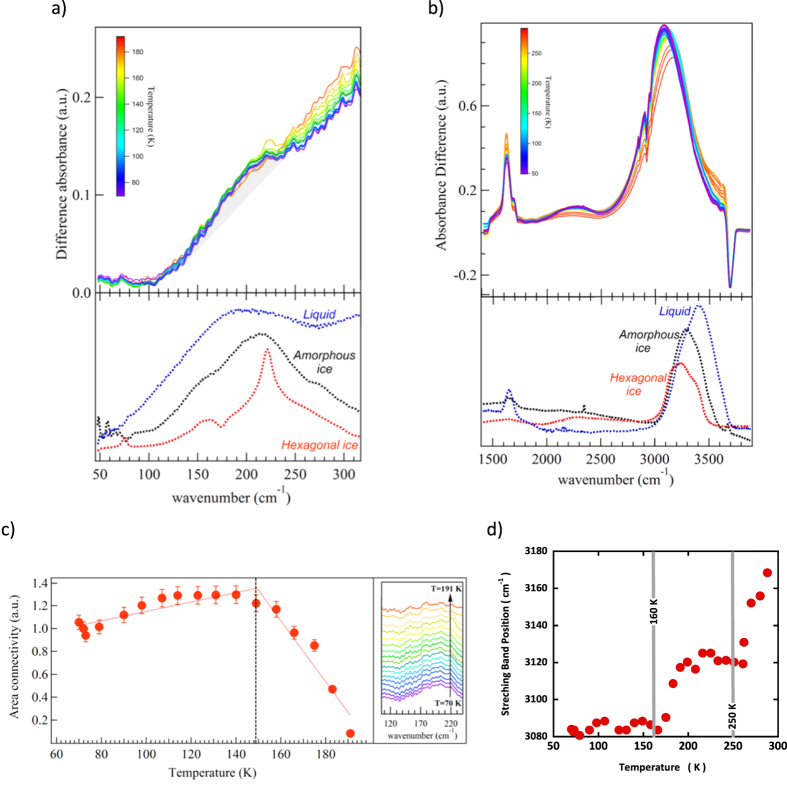
Temperature dependence of FIR (**a**) and MIR (**b**) spectra of interfacial water from 80 to 300 K. In each case, the signal of hexagonal or amorphous ice (LDA) and liquid water are shown for comparison. The weak and broad band around 210 cm^−1^ shown in (**a**) is the connectivity band. It is superimposed to the libration band starting at 250 cm^−1^. (**c**) Integrated intensity of the spectra shown in the inset on the right. These are the difference spectra (grey area shown in (**a**) of the FIR connectivity band and a linear background accounting for the libration band. (**d**) Position of the maximum of the MIR stretching band as a function of temperature.

**Figure 4:**
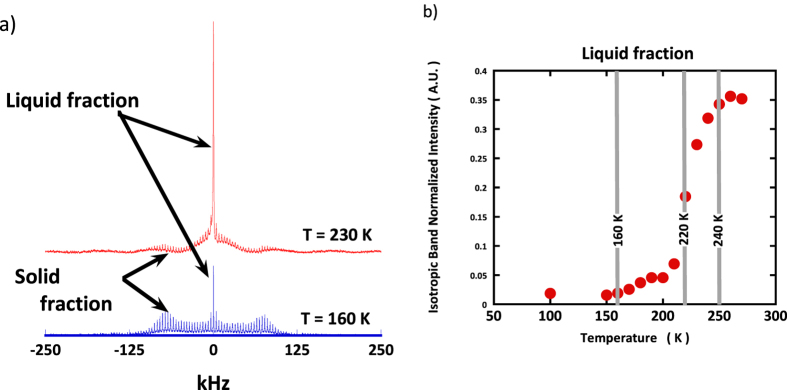
(**a**) SS-NMR spectra of water D_2_O monolayer in Vycor at 160 and 230 K. The broad Pake pattern at c.a. 1000 ppm is to be inferred to a fraction of molecule with no mobility (solid-like), while the central narrow band at 0 ppm is due to a fraction of molecules experiencing rapid reorientational dynamics (liquid-like). (**b**) Evolution as a function of temperature, of the fraction of mobile (liquid-like) water molecule, f, estimated from the relative intensity of the central isotropic line as a function of temperature.

**Figure 5:**
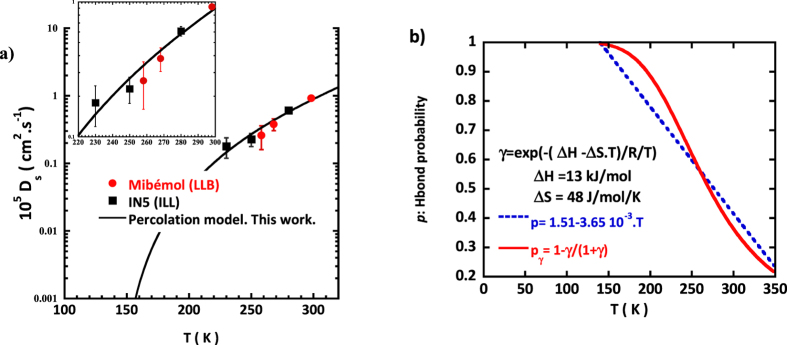
(**a**) To tune, p, the probability to form a HBond, we use the temperature dependence of the self-diffusion coefficient of a monolayer of water on Vycor (Eq. 5) as measured by QENS (Spectrometers Mibémol at LLB, Saclay and IN5 at ILL, Grenoble). A good agreement (a zoom is shown in inset) is reached for a first-order approximation: p = 1.51–3.65 10^−3^.T. (**b)** Considering the HBond network structure as resulting from the dynamical equilibrium between broken and intact HBonds, one can estimate the enthalpy and entropy associated to the formation of a HBond. In this case p_γ_, the probability for a HBond to be formed is not linear (Eq. 8) and an estimate of the temperature dependence of the interfacial water specific heat can be computed (Eq. 9). The calculated *C*_*p*_ temperature dependence is shown on Fig. 2.

The interpretation of the enthalpy value is more subtle: a single HBond with a silanol surface group of an interfacial water molecule on Vycor is stronger (see the position of the MIR OH stretching mode Fig. 3b) than a HBond in bulk, but the probability to deal with an intact bond being smaller, after thermal average, the average HBond strength is smaller than in bulk. We point-out that such a softer HBond strength in interfacial water is consistent with the experimental observation that interfacial water remains liquid above 160 K (Fig. 4b). This conclusion is also in line with the conclusion derived from the MIR data (Fig. 3d) that the HBond becomes “softer” when temperature increases above 160 K.

### Robustness of the model: temperature dependence of interfacial water specific heat

In bulk, the large specific heat of water, that allow its good heat transfer property, is a direct consequence of the energy needed to disrupt HBonds. In the framework of the present model of interfacial water, the temperature dependence of the interfacial specific heat can be directly estimated as the temperature derivative of the probability to deal with an intact bond:


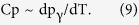


As shown on Fig. 2, the model presented here provides a qualitative agreement with the global shape of the experimental *C*_*p*_ measurement^24^. In particular it reproduces well the experimental increase of the *C*_*p*_ values above 160 K. This *C*_*p*_ increase is indeed due to the gradually increase of the water molecules dynamics in the system (Fig. 4b and Supplementary Fig. S1).

## Discussion

We consider the two key interactions at play when it comes to water *i)* the hydrogen bond at the local level and *ii)* the extension of the HBond network at the mesoscopic scale. The essence of the approach developed here is to point-out that the local density around a given water molecule is ruled by the number of short-lived HBonds this molecule experiences. Two extreme situations can be described: the presence of a molecule engaged in four HBond induces a local low *ice-like* density, while the density is maximum if the molecule is not bound to any neighbouring molecule.

Compared to bulk water, where such a zero-HBond situation is possible, the specificity of the 2D situation is to consider that a water molecule is permanently engaged in at least one HBond: the one between the “tagged” water molecule and the hydrophilic surface.

As the HBond is a transient interaction (typically a few ps at room temperature), an assembly of water molecules experiences constant density, hence entropy, fluctuations.

We account for the fluctuating HBond network with a simple and purely analytical mean-field percolation model: from experimental self-diffusion coefficients data, we estimate the temperature dependence of the probability for a HBond to be formed. We then focus on the statistics of the four coordinated water molecules.

We are able to derive the average entropy and enthalpy of interfacial water. The entropy (Δ*S* *=* 48 J/mol/K) is found to be twice the one of bulk water. Our interpretation is that the two dimensional topology frustrates the optimal perfect tetrahedral geometry easily reached in bulk (hexagonal ice, Fig. 1a). As a consequence, even at temperatures as low as 160 K, the thermal energy is sufficient to break a few HBonds. Even if the correlation time is very long (slow glassy-like dynamics), the molecules can then reorient and find the conformation of minimal energy i.e. form 4 HBonds. In the framework of the percolation view of the HBond network, the fraction of water molecules engaged in 4 HBonds are not distributed at random but form patches (Fig. 1c). At 160 K, once a few molecules experience 4 HBonds (even imperfect on a geometrical point of view) they tend to clamp together.

**Figure 6:**
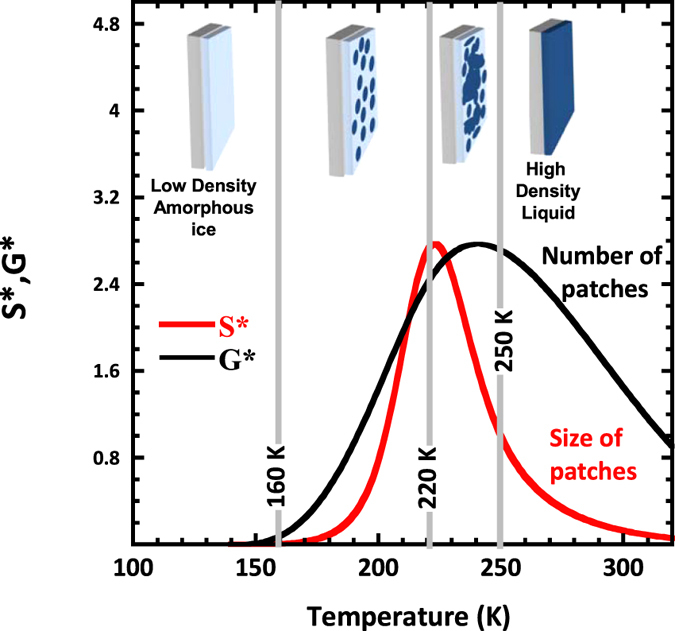
By injecting the temperature dependence of p, the probability to form a HBond (Fig. 5b), in Eqs 2 and 3, we estimate the temperature dependence of S* and G*, respectively the size and the number of patches of water molecules all engaged in 4 HBonds (these patches show a low density). Within the 2D percolation model, we focus on the patches of 4 HBonded molecules (one HBond engaged with the surface and three with neighbouring water molecules) and propose a global interpretation, in terms of water molecules connectivity, of the three dynamical crossovers experimentally observed,: i) at 160 K, the thermal energy of the system is large enough to induce the breaking of few HBonds. The system experiences a glass transition temperature and dynamical, although rare and associated with long correlation times, events become possible ii) at 220 K a percolation of the high density patches formed above 160 K occurs while iii) at 250 K their number and hence the density fluctuations are maximum, inducing a liquid-liquid transition. The four sketches, reminiscent of Fig. 1c, shown on top of the figure provide snapshots view (in reality, the structure is a dynamical equilibrium) of the different mesoscopic structures of the water monolayer. The Vycor substrate is represented in grey, the light blue area stands for low density and the dark blue for the high density liquid fraction. As classically in the field of percolation, the infinite cluster formed at 250 K is not considered in the S*statistics.

The model presented here provides then a sound and clear interpretation of the different events experimentally (neutron, NMR and infra-red spectroscopy) detected in interfacial water at 160, 220 and 250 K. Starting from a low density amorphous state of water at low temperature, these transitions are respectively interpreted as the onset of appearance of transient patches of 4-HBonded molecules at 160 K. By increasing the temperature, these *nuclei* of 4 HBonds *molecules* become more likely (G* increase, Fig. 6) and grow in size (S* increase, Fig. 6). At 220 K, these domains percolate and finally totally cover the surface at 250 K. This picture provides a qualitative explanation for the, so far unexplained, broad transition at 160 K detected in the specific heat of interfacial water, but also for the significantly shift of the *C*_*p*_ to a larger value compared to the bulk water (i.e. ice below 273 K).

Although Vycor, with its perfect non fluctuation and homogeneous surface and structure, can be considered as a pure model system for physicist, we think that the temperature transitions of 2D water we report in this paper have a general relevance. For example, in the field of biophysics, Mazza *et al*.^25^ report on an HBond network of a percolating layer of water molecules on the surface of a soluble globular protein (Lysozyme). They observe by dielectric spectroscopy protons relaxations modes with two cross-overs at approximately 180 and 250 K. These experimental transitions are consistent with a coarse-grained and Monte Carlo model of hydration water ((extension of the present work with MD simulation is discussed in SI text 6) that shows two maxima in the specific heat. At 180 K, the transition and associated specific heat maximum is attributed to a maximum of fluctuation of the cooperative local reordering of the HBond network while at 250 K, it is associated to the maximum fluctuation of the formation and breaking of HBonds. Our present work that reveals the coexistence of two form of water in this 180 and 250 K range provide an original interpretation of these important results.

### Relevance to the debate on the existence of a low temperature critical point in water

Since it is a notorious way to avoid nucleation, nanometric confinement of water has effectively been intensively used to probe the liquid phase in this 150 to 235 K “No man’s land”. A HDL to LDL transition has been indeed on the focus over the last decade as such a transition line is expected to end by a critical point. Indeed, an evidence of such a critical point would be a first level discovery as it would lead to a revolutionary re-interpretation of the physical properties of bulk water.

But, the often reported 220 K Fragile to Strong Transition (another wording for the LDL/HDL transition) of water in extreme confinement comes to a situation where 2–3 water layers in between pore walls are considered. We show in this paper such a 220 K transition in a single monolayer of water. We conclude that, as it reveals the prominently underlying properties of the interfacial water in the 220–250 K region, this confinement strategy cannot bring any firm conclusion about the physics of bulk water and in particular the existence of the low temperature critical point. It should nevertheless be noted that MD simulations suggest that the local geometry of the HBond could drive the existence^26^ of the low temperature critical point. The present case of 2D water, where the large entropy suggest that, compared to the ideal tetrahedral geometry, we are dealing with significantly distorted HBonds is indeed a situation unfavourable for the existence of the low temperature critical point.

## Conclusion

This paper brings together two scientific hot topics: water physics and physics in two dimensions^27^. We account for the transient and fluctuating HBond network of 2D water by a simple and purely analytical mean-field percolation model. We show that, while simple, this model is robust enough to account in a coherent manner for a whole set of experimental structural, dynamical and thermodynamical data on an extended temperature range from 150 to 300 K.

So far, most of the studies of water under confinement or interfacial water have concluded to the existence of two liquid forms of water: a consensus has emerged that a low density liquid (LDL) at low temperature turns into a high density liquid (HDL) in the 220 K region. We also conclude to the existence of these two forms of water. But we provide a new insight: instead of a simple LDL to HDL transition, we show that 2D water is a heterogeneous system characterized by the coexistence of the LDL and HDL forms between 160 and 250 K. We show that the equilibrium between low and high-density transient clusters is controlled by the temperature and that the high density liquid patches coalesce at 220 K. At this temperature, this infinite HDL cluster coexists with non-percolating LDL patches.

This strong heterogeneous nature of interfacial water may have important implications in the interpretation of the physics of systems where low temperature interfacial water is at play: in biophysics for example where the protein 220 K dynamical transition is strongly correlated with enzymatic activity or photosynthetic processes, and generally in all systems where water participates to the stabilization of the surface of biological systems^28^.

## Material and Methods

### Sample preparation

Vycor^®^ (Vycor brand porous glass n° 7930 is a product of Corning Glass Works) is made of a SiO_2_ open porous network with a characteristic size of 50 Å and an interface of 130 m^2^/g specific surface area^29^,^30^. The numerous silanol (Si-OH) groups covering its surface make the Vycor a very hydrophilic material. It can for example easily absorb water up to 25% of its dry mass. This is fully (100%) hydrated Vycor. Partially hydrated samples can also be prepared by absorption of water in the vapour phase. In this paper, we specifically consider two extreme samples: *i)* fully hydrated Vycor (corresponding to the mass ratio x_m_ = m_water_/m_dry Vycor_ = 0.25) and *ii)* low hydrated sample (x_m_ = 0.06 also referred as 25% hydration). In the later, water realizes monolayer coverage of the Vycor surface and will be referred to, hereafter, as interfacial water (IW). Vycor has been hydrated with D_2_O for SS-NMR and quasi-elastic neutron scattering experiments respectively. See SI text for details about the methods used for the IR, NMR and specific heat measurements.

## Additional Information

**How to cite this article**: Zanotti, J.-M. *et al*. Competing coexisting phases in 2D water. *Sci. Rep.* **6**, 25938; doi: 10.1038/srep25938 (2016).

## Supplementary Material

Supplementary Information
