# Supplementary material for: Competing coexisting phases in 2D water
Source: Sci Rep. 2016 May 17;6:25938. doi: 10.1038/srep25938 (PMC4869029; doi:10.1038/srep25938)
Supplement: Supplementary Information [file srep25938-s1.pdf]

SUPPLEMENTARY INFORMATION

Competing coexisting phases in 2D water

Jean-Marc Zanotti[1*], Patrick Judeinstein[1,2], Simona Dalla-Bernardina[3], Gaëlle Creff[3], Jean-Blaise Brubach[3], Pascale Roy[3], Marco Bonetti[4], Jacques Ollivier[5], Dimitrios Sakellariou[6], and Marie-Claire Bellissent-Funel[1]

1- Reminder about the Gibbs Thomson Effect:

The Gibbs-Thomson effect is directly related to *i)* the significant contribution of a term related to the solid-liquid interface in the Gibbs-Duhem chemical equilibrium relation (in bulk this interfacial contribution is neglected) and *ii)* to the existence of a surface curvature imposed by the confining geometry. This is formalized by the expression: $\Delta T_m = (4.\ \sigma_{sl} / \Delta H_f / v).T_m/d$, where ΔH_f is the bulk enthalpy of fusion, v is the molar volume of the solid fraction, σ_{sl} the surface energy of the solid-liquid interface, T_m the bulk melting point and d the pore diameter. It clearly appears that, ΔT_m, the downshift of the melting temperature is inversely proportional to the characteristic size of the porous structure.

2- Already available data on low temperature interfacial water mobility as revealed by QENS derived mean-square-displacement:

The dynamics of interfacial water has already been analysed in details by neutron diffraction and quasi-elastic neutron scattering. In QENS, in a way similar to the Debye-Waller effect, the Q dependent Gaussian intensity loss $exp(-Q^2.\langle u^2\rangle)$ can be related to the spatial extension ($\langle u^2\rangle$) of atoms around their equilibrium position (also referred as Mean-Square Displacement, MSD). Furthermore, the exact Q dependence of the neutron intensity loss enables to assess, $\langle u^2\rangle_{Rot}$ and $\langle u^2\rangle_{Trans}$, the MSD due to respectively the rotational and translational modes of the water molecules[1].

QENS data as a function of temperature indicate[2] (see Supplementary Fig.S1), that on a 1 nanosecond timescale:

(i) Below 155 K, interfacial water is a Low Density Amorphous (LDA) ice (with $Q_0=Q_0^{LDA}=1.71$ Å^{-1} the first peak in *S(Q)* structure factor, see Fig.S1.b) showing no long range translational dynamics ($\langle u^2\rangle_{Trans}\approx 0$, see Fig.S1.g). Above 160 K, no change is observed in water density (Fig.S1.b) but translational dynamics becomes possible on a 1 nanosecond timescale, as shown by the $\langle u^2\rangle_{Trans}$ non null value (Fig.S1.g). This is a clear signature of a glass transition.

(ii) On increasing the temperature $\langle u^2\rangle_{Trans}$ and $\langle u^2\rangle_{Rot}$, increases linearly (Fig.S1.f and g, this means that the related correlations times decrease). At 220 K, however, the correlation times associated to $\langle u^2\rangle_{Rot}$ becomes suddenly more temperature dependent.

(iii) At 250 K, this Low Density Liquid (LDL) experiences a transition to High Density Liquid HDL ($Q_0=1.86$ Å^{-1}, Fig.S1.b) and rotational and translational correlation times related to $\langle u^2\rangle_{Trans}$ and $\langle u^2\rangle_{Rot}$ significantly decrease and become shorter than the accessible time window of the spectrometer (1 ns).

3- The interfacial water molecules engaged in 4 HBonds are not randomly distributed but form patches:

Let's give a numerical example by considering *i)* a central water molecule, *ii)* its four immediate neighbours and *iii)* that the probability to form a HBond is p=0.5. If nothing is known about the connectivity of the neighbours, the probability that this central molecule is HBonded to its neighbours is p^4 = 0.5^4 ~ 0.06. But now, if we know that each of the four neighbouring molecules are themselves engaged in four HBonds with their own neighbours, then, the probability that the central molecule is itself engaged in four HBonds is 1. In other words, if each of the four neighbours of a given water molecules has all four intact HBonds (i.e. belongs to the class f_4), this molecule also belongs to the f_4 class[3].

4- Derivation of the number (G*) of 4 HBonded water molecules patches and size (S*):
The equations 2, 3 and 4 are directly derived from the classical analytic results of Flory[4] to calculate the distribution of molecular sizes of three dimensional polymeric gelation reactions. Flory defines a parameter f as the functionality *i.e.* the number of bonds a monomer can form with other monomers. The direct parallel between gelation and the extension of the HBond network in the case of bulk water has been proposed by Stanley et al.[5] . A functionality f=4 naturally accounts for the case of bulk water. In the case of 2D water, as only 3 HBonds are available for the extension of the network, in this work, to obtain Eq.4, we use f=3.

5- Self-diffusion coefficient as measured by QENS can be used to tune the temperature dependence of *p*, the probability to form a hydrogen bond:
In water, translational motion of molecules is only possible when several HBonds are simultaneously broken[1]. Rotational motion therefore controls the long range translational diffusion of molecules and the relation $\tau_{Rot}.D_s = C_1$, where C_1 is a constant and τ_{Rot} is the rotational relaxation time, holds[6].
If one considers that a water molecule engaged in 2, 3 or 4 HBonds it is too strongly linked to diffuse away, diffusional motion is only possible for molecules with zero or a single intact HBond, i.e. belonging to the f_0 or f_1 families. The fraction of immobile molecules is then $F_{Im}=f_2+f_3+f_4$:

$$F_{Im} = \sum_{i=2}^{4} \binom{3}{i} p^2(1-p)^{3-i} = 3p - 3p^2 + p^3 \qquad \textbf{(SE 1)}$$

By principle, a percolation model as the one we use provides only static quantities. As suggested by Stanley and Teixeira[3] a way to introduce time dependant quantities in such a model is to consider that the elemental time pacing the time evolution of the structure of an assembly of water molecules is τ_{HB}, the characteristic life time of an HBond. If one observes the systems with n successive snapshots each separated by a time τ_{HB}, the probability that a randomly chosen molecule has not diffused away (*i.e.* has remained immobile) is F_{Im}^n. If after these n successive realizations, the molecule adopts a rotational motion, the duration $n.\tau_{HB}$ is a good estimate of the rotational relaxation time τ_{Rot}. One can then pose $F_{Im}^{\tau_{Rot}/\tau_{HB}} = C_2$, where C_2 is a constant. The self-diffusion coefficient then writes:

$$D_s = C.ln(F_{Im}) = C.ln(3p - 3p^2 + p^3) \qquad \textbf{(SE 2)}$$

6- Possible extension of this model/study by molecular dynamics simulation:

As the most recent developments on the physics of water are mostly based on MD results, we think that the simple and physical appealing model proposed here, shows a noticeable originality. We nevertheless note that a MD approach would be particularly adapted for estimating the precise contributions of the two or three HBonds "patches" neglected here. MD would be indeed a precious tool to test what we think is the essence of the 2D water physical behaviour: the spectacular excess of entropy (twice the one of bulk water, see Fig.5 and related discussion) of water molecules induced by the reduced occurrence of stable hydrogen bond (in particular the O-H---O linearity[1]), imposed by the 2D topology.

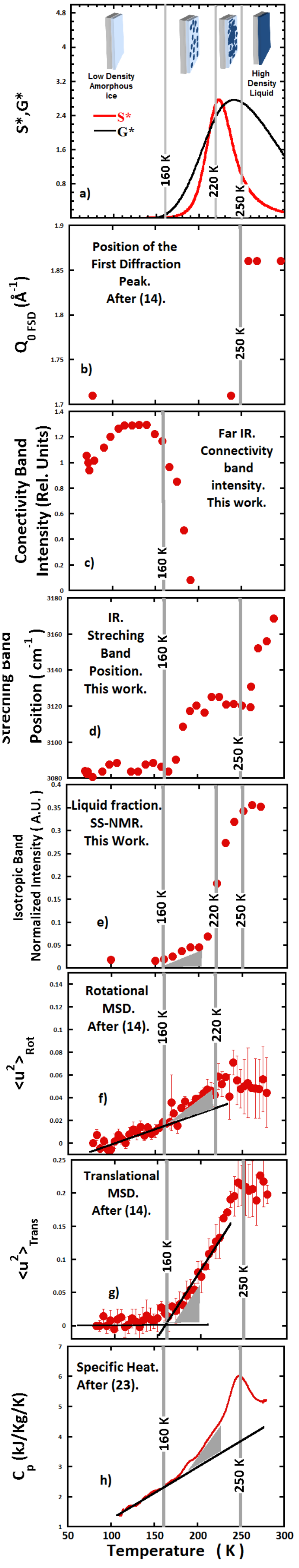

Supplementary Fig.S1. *Synoptic view of the temperature dependence of available experimental quantities of 2D water as a function of temperature. For the sake of comparison the top figure a) reproduces the Fig.6 to show along with the experimental data the size (S*) and the number (G*) of the high density liquid patches derived from the percolation model.*

The specific heat of a material is the frequency weighted integration of the number of its dynamical modes. It is also a probe the degree of density fluctuations the system experiences. The grey area colored on Fig. e) f) g) and h) highlight the concomitance of dynamical modes related quantities (MSD and fraction of liquid) of interfacial water molecules as measured by SS-NMR and neutron scattering, with the onset of specific heat excess above 160 K. The sudden change in the FIR connectivity band (Fig.c) intensity at this temperature indicates a change in the intermolecular collective dynamics of clusters of water molecules. This change has an incidence on the MIR intramolecular stretching mode (Fig.d). Our interpretation is that at 160 K, the thermal energy is sufficient to break few distorted HBonds so that few molecules of a LDA form of ice (Q_0=1.71Å^{-1}, Fig.b) can experience enough dynamics to collapse in few small patches of mobile high density liquid patches. We assign the 160 K events to a glass transition of interfacial water.

At 220 K the sudden increase of the fraction of liquid-like water as detected by SS-NMR (Fig.e) comes along with a saturation (due to instrumental limitation see reference [2]) of the MSD due to the water rotational modes (Fig.f). Our interpretation is that, at this temperature, the high density liquid patches formed above 160 K have increased in both number and size (Fig.a) so that when they percolate the surface accessible to the molecules suddenly significantly increases hence their MSD.

At 250 K, the high density liquid patches have grown up and multiplied so much (Fig.a) that the high density liquid totally invades the surface: both the fraction of liquid (Fig.e) and its translational MSD (Fig.f) become resolution limited. The water density, as inferred from the position of the neutron diffraction first sharp diffraction peak (Fig.b) and MIR stretching mode tend to recover the values of water, but somehow supercooled. This is a real thermodynamical event (we interpret as a liquid-liquid transition) well detected in calorimetry (Fig.h).

This figure S1 shows a global trend: the reality of the experimental transitions we describe at 160, 220 and 250 K and their coherence with our interpretation within the surface percolation model we propose in this paper.

Supplementary Material and methods:

Infra-Red Spectroscopy:

Measurements were performed at the AILES beamline with a Bruker IFS125 FT-IR spectrometer using synchrotron far and mid infrared radiation emitted at SOLEIL facility (Saint-Aubin, France). Spectra from 20 cm^{-1} to 5000 cm^{-1} were obtained by averaging 200 scans. In the far infrared region (FIR: 20 cm^{-1} to 600 cm^{-1}) spectra were recorded using a 6 µm thick Mylar beam splitter and a bolometer with a resolution of 2 cm^{-1}, while in the mid infrared region (MIR: 600 cm^{-1} to 5000 cm^{-1}) they were recorded using a KBr beam splitter and a MCT detector with a resolution of 4 cm^{-1}.

A specific homemade cell[7] was used to measure hydration/dehydration processes *in situ.* This cell is equipped with two diamond windows to allow absorbance measurements (leak rate <10^{-9} mbars·l/s). Vycor sample (thickness of ~80 μm) was placed inside the cell, perpendicularly to the incident beam with both surfaces exposed to the humidified air/pumping allowing the hydration/dehydration processes. Sample was hydrated by H_2O. As described elsewhere[7] sample temperature is controlled by a cryostat (precision 0.2 K) while sample hydration is monitored by a thermostated gauge (0-100 mbar with a precision of 0.02 mbar). The gauge provides control of the water vapor pressure (*p)* in the cell. The relative humidity (RH) to which Vycor is exposed can be precisely set to the value (p/p_0)*100, p_0 being the water vapor pressure equal to 28.1 mbar at 23°C. By setting the pressure at 9.1 mbar we were able to prepare Vycor equilibrated at 32.4% RH. Firstly, spectra of the dried sample from T=70 K to T=298 K were acquired to be used as a reference. Then, spectra of the sample in equilibrium with 9.1 mbar of water vapor pressure were recorded at the same temperatures. Difference spectra are displayed in absorbance units as $-log(I/I_0)$, where *I* and I_o are respectively the hydrated sample and dried sample transmissions at the same temperature.

NMR

Solid state Magic Angle Spinning NMR (SS-NMR) experiments were performed on a Bruker AVANCE II spectrometer operating at 11.74 T. Spectra were recorded at 76.8 MHz and spinning rate of the rotor was ajusted to 4 kHz. The Vycor sample was hydrated by D_2O and packed inside a 7 mm outer diameter Bruker rotor. A liquid nitrogen dewar was used to provide gas for bearing and drive and the temperature of the sample was set by the Bruker temperature control unit. Special care was put to avoid freezing the bore of the magnet, and users should be extremely careful.

Specific heat measurements

Specific heat measurements were performed using quasi-adiabatic AC calorimetry where the sample is heated periodically at f_0=10 mHz and the corresponding temperature oscillation is measured at $2f_0$ around the mean temperature of the reservoir. Slices of Vycor 2 mm thick and 12 mm in diameter soaked with water were housed in a leak-proof small silver cell enclosed in a cryostat cooled by liquid nitrogen. Specific heat measurements were performed by heating the cell at rate of ≈1mK/s in the temperature range 113 to 373 K after cooling the sample at a rate between 1 and 2 K/min.

1. In bulk liquid water, a molecule is hydrogen bonded to on average slightly less than four neighboring molecules. If due to thermal energy, a hydrogen bond (HBond) O---H-O moves apart from linearity by more than 25°, the bond breaks. When several H-bonds engaged by a molecule are simultaneously broken, the molecule is free to experience a rotational diffusive motion until several hydrogen bonds are formed again leading to the formation of a transient localization “site”. Indeed, instead of addressing an *average* water dynamics, considerable insight is obtained by individually considering the rotational and translational modes to the overall dynamics.

2. Zanotti J.-M., Bellissent-Funel M.-C., Chen S.-H. Experimental evidence of a liquid-liquid transition in interfacial water. Europhys. Lett. **71**, 91–97 (2007).
3. Stanley H.E. and Teixeira J. Interpretation of the unusual behavior of H_2O and D_2O at low temperatures: Tests of a percolation model. J. Chem. Phys. **73**, 3404-3422 (1980).
4. Flory P.J. Molecular size distribution in three dimensional polymers. I. Gelation. J. Am. Chem. Soc. **64**, 3083–3090 (1941).
5. Stanley H.E., Blumberg R. and Geiger A. Gelation models of hydrogen bond networks in liquid water. Phys Rev *B,* **28**, 1626–1629 (1983).
6. Pruppacher H.R. Self-Diffusion Coefficient of Supercooled Water. J. Chem. Phys. 56, 101 (1972).
7. Dalla Bernardina S., Alabarse F., Kalinko A., Roy P., Vita N., Hienerwadel R., Berthomieu C., Judeinstein P. , Zanotti J.-M., Bantignies J.-L., Haines J., Catafesta J., Creff G., Manceron L., Brubach J.-B. Vibrational Spectroscopy New experimental set-ups for studying nanoconfined water on the AILES beamline at SOLEIL. Vib Spectrosc 75, 154–161(2014).
